# Mitochondrial transport serves as a mitochondrial quality control strategy in axons: Implications for central nervous system disorders

**DOI:** 10.1111/cns.13122

**Published:** 2019-03-21

**Authors:** Yan‐Rong Zheng, Xiang‐Nan Zhang, Zhong Chen

**Affiliations:** ^1^ Institute of Pharmacology and Toxicology, NHC and CAMS Key Laboratory of Medical Neurobiology, College of Pharmaceutical Sciences Zhejiang University Hangzhou China; ^2^ Department of Neurology, Second Affiliated Hospital, School of Medicine Zhejiang University Hangzhou China

**Keywords:** central nervous system disorders, mitochondrial dynamics, mitochondrial transport, mitophagy

## Abstract

Axonal mitochondrial quality is essential for neuronal health and functions. Compromised mitochondrial quality, reflected by loss of membrane potential, collapse of ATP production, abnormal morphology, burst of reactive oxygen species generation, and impaired Ca^2+^ buffering capacity, can alter mitochondrial transport. Mitochondrial transport in turn maintains axonal mitochondrial homeostasis in several ways. Newly generated mitochondria are anterogradely transported along with axon from soma to replenish axonal mitochondrial pool, while damaged mitochondria undergo retrograde transport for repair or degradation. Besides, mitochondria are also arrested in axon to quarantine damages locally. Accumulating evidence suggests abnormal mitochondrial transport leads to mitochondrial dysfunction and axon degeneration in a variety of neurological and psychiatric disorders. Further investigations into the details of this process would help to extend our understanding of various neurological diseases and shed light on the corresponding therapies.

## 1 INTRODUCTION

The disorders of central nervous system could be devastating due to failures in controlling basic physiological functions and problems with emotional changes and mental tasks. In the past decades, extensive efforts have been made to develop therapies against central nervous system disorders by targeting ion channels and neurotransmitters.^1^, ^2^ Besides these achievements, insights gained in recent years have revealed the critical contribution of mitochondria to neuronal health.^3^ Mitochondria not only serve as the “powerhouse” of neurons but also play essential roles in metabolizing neurotransmitters, buffering Ca^2+^, and sending signal deciding neuronal survival.^4^ Mitochondrial quality is therefore the basis of neural homeostasis. Stress‐induced mitochondrial damage results in the collapse of bioenergy generation and even worse, neuronal demise.^5^, ^6^ Neurons monitor mitochondrial quality by replenishing mitochondria, repairing damaged mitochondria by fusing with healthy ones, or eliminating mitochondria through autophagic clearance (mitophagy). The dysfunction of mitochondrial quality control has been associated with various neurological disorders.^5^, ^6^, ^7^, ^8^

Highly polarized morphology distinguishes neurons from other cells. Their long, thin axons are not only unique in morphology but also fundamental to neuronal functions by forming specialized compartments such as synapses and nodes of Ranvier. To meet the ever‐changing demands of neurons, mitochondria have been located in these distal compartments in axons.^4^ However, it is more challenging for neurons to control the quality of these distal mitochondria due to the limited extent of both mitochondrial biogenesis and degradation in axons.^9^ To cope with this, neurons develop finely regulated transport system. Specifically, mitochondria newly generated in soma are distributed to axons by anterograde transport, while senescent or damaged mitochondria in axons undergo retrograde transport for repair or autophagic clearance.^9^ Mitochondria are distributed according to their quality, which is fundamental to neuronal homeostasis and functions.^4^ The alternation of axonal mitochondrial transport is intimately related to compromised mitochondrial quality and thus contributes to neurological disorders. For example, aberrant protein aggregates and disease‐relevant mutants are prone to compromise axonal mitochondrial trafficking, which further leads to mitochondrial dysfunction and neuronal death in a number of neurodegenerative disorders.^10^, ^11^, ^12^ Therefore, correction of disrupted mitochondrial transport seems to be a promising therapy for some neurological disorders. Here, we review the contributions of mitochondrial transport to axonal mitochondrial quality control and its implications for neurological disorders.

## 2 MITOCHONDRIAL TRANSPORT MACHINERY

Mammalian axons have polarized microtubules with their plus ends oriented toward the terminus and minus ends toward the soma.^13^ In general, two kinds of motor proteins are responsible for transporting mitochondria in two directions, that is, kinesin family for anterograde transport (away from soma) and dynein‐dynactin complex for retrograde transport (toward soma). In addition, specific mitochondrial anchor protein takes responsibility for mitochondrial docking in axons.

### 2.1 Anterograde transport motors

Among the kinesin family members, kinesin‐1 (also known as KIF5) serves as the major motor for the anterograde transport of neuronal mitochondria.^14^ Kinesin heavy chain (KHC) interacts with mitochondria via adaptor proteins, Miro and Milton. Miro is an atypical Rho (Ras homolog) family of GTPases which locates in outer mitochondrial membrane (OMM). Miro interacts with Milton which binds to the C‐terminus of KHC. Mutations in either Miro or Milton disrupt mitochondrial anterograde transport in axons.^15^, ^16^ Besides Miro and Milton, syntabulin also adapts KHC to mitochondria.^17^ In addition, hypoxia‐up‐regulated mitochondrial movement regulator (HUMMR) senses hypoxia and interacts with Miro to regulate mitochondrial mobility.^18^ Some other kinesin motors, such as kinesin‐3 family proteins KIF1Bα^19^ and KLP6,^20^ may also drive anterograde mitochondrial motility.

### 2.2 Retrograde transport motors

Cytoplasmic dynein drives the mitochondrial retrograde movement in axons. Unlike kinesin family, there is only one identified dynein motor. Dynein is proposed to form a complex with dynactin, and the complicated structure of dynein‐dynactin complex makes it hard to study. Loss of Miro reduces both anterograde and retrograde mitochondrial transport in Drosophila,^21^ implying the involvement of Miro/Milton in mitochondrial retrograde transport as adaptor proteins. Supportively, dynein‐dynactin complex has been shown to interact with Miro and Milton.^22^

### 2.3 Anchor protein

Syntaphilin serves as a mitochondrial anchor protein in axons through its axon‐sorting domain and microtubule‐binding domain.^23^ In addition, SNPH‐mediated mitochondrial docking is also dependent on kinesin^24^ and dynein light chain LC8.^25^ A recent study indicated the recruitment of syntaphilin to mitochondria by optogenetic approach was sufficient to arrest mitochondria transported promptly in both directions. It seems that motor and anchor proteins present on one particular mitochondrion simultaneously and the balance of their forces may decide the direction of mitochondrial movements.^26^

## 3 MITOCHONDRIAL QUALITY AND CHANGES OF MITOCHONDRIAL MOVEMENTS

Mitochondrial transport responds to mitochondrial quality and thereby modulates their distribution, which is important to neuronal functions and homeostasis. Compromised mitochondrial quality can be reflected by loss of membrane potential, collapse of ATP production, abnormal mitochondrial morphology, burst of mitochondrial reactive oxygen species (ROS), and impaired capacity of Ca^2+^ buffering. Here, we discussed how impaired mitochondrial quality may impact on mitochondrial trafficking.

### 3.1 Mitochondrial membrane potential

Stresses or aging can result in the loss of mitochondrial membrane potential, which is also defined as mitochondrial depolarization. Under physical conditions, axonal mitochondria with normal membrane potential are transported anterogradely and those with low membrane potential are transported toward the cell body.^27^ However, mitochondrial uncoupler carbonyl cyanide 3‐chlorophenylhydrazone (CCCP) induces the phosphorylation and degradation of Miro in a PINK/Parkin‐dependent pathway,^28^, ^29^ which further reduces mitochondrial mobility in rat hippocampal axons.^28^ In addition, as illustrated by a Friedreich ataxia model in *Drosophila*, depolarized mitochondria with normal mitochondrial ROS (mtROS) production have shown deficits in axonal trafficking, implying mitochondrial depolarization is sufficient to arrest mitochondria.^30^

### 3.2 ATP production

Cargo transportation along the axon is an ATP‐consuming process, and more intriguingly, motors for mitochondria seem to be fueled by ATP generated from oxidative phosphorylation rather than glycolysis.^31^ In cortical neurons from rat brains, inhibition of mitochondrial ATP production by mitochondrial H^+^‐ATP‐synthase inhibitor oligomycin arrests axonal mitochondrial trafficking while the inhibition of glycolysis shows little impact on mitochondrial mobility.^31^ These results indicate proper mitochondrial energy generation is the basis for mitochondrial movements in axons and bioenergetic crisis caused by aging or stresses^32^ may undermine axonal mitochondrial trafficking. On the other hand, axonal mitochondrial mobility changes to fit the neuronal metabolic demands. High extracellular glucose level also decreases axonal mitochondrial mobility by O‐GlcNAcylation of Milton,^33^ which in turn improves the availability of glucose to mitochondria and thus facilitates bioenergy generation.

### 3.3 Mitochondrial morphology

Mitochondrial morphology is regulated by several mitochondrial fission and fusion proteins. Dominant negative or depletion of mitochondrial fusion protein mitofusin 2 (Mfn2) causes mitochondrial fragmentation, which coincides with disrupted mitochondrial movement in axons.^34^ Furthermore, both Mfn1 and Mfn2 interact with Miro and Milton through which they regulate axonal mitochondrial transport.^34^ Additionally, dynamin‐related protein 1 (Drp1) not only serves as a mitochondrial fission protein but functions with actin‐related protein 10 (Actr10) to promote mitochondrial retrograde transport in axons.^35^ However, mitochondrial morphology may not be the primary factor to drive mitochondrial trafficking, since the abundance or activity of mitochondrial fission/fusion proteins could be affected by mitochondrial membrane potential loss,^36^ ATP depletion,^37^ and mtROS generation.^38^

### 3.4 Mitochondrial reactive oxygen species

Although mitochondria are the major sites for intracellular ROS generation,^39^, ^40^ the association of ROS with axonal mitochondrial transport has been poorly understood. In nonneuronal cells, mitochondria may cause accumulation of nuclear ROS after their retrograde transport during hypoxia.^41^ The majority of ROS triggered by hypoxia originate from mitochondrial complex III.^42^ Consistently, mild and prolonged incubation of complex III inhibitor antimycin A promotes axonal mitochondrial retrograde transport in hippocampal neurons.^43^ In addition, ROS seem to increase the ATPase activity of dynein.^44^ However, ROS caused by extracellular H_2_O_2_ incubation arrest axonal mitochondrial mobility both in vitro^45^ and in vivo.^46^ It remains undetermined whether different sources or abundance of ROS exert on mitochondrial trafficking distinctively. Neurons undergoing ischemia may provide a model to address this issue, in which mtROS^40^ and mitochondrial quality control play critical roles.^7^, ^47^, ^48^, ^49^

### 3.5 Mitochondrial calcium buffering

Mitochondria have also been deemed critical for neuronal Ca^2+^ buffering. Although it is well established that cytosolic Ca^2+^ is a key regulator of mitochondrial transport, the association of mitochondrial Ca^2+^ with their movement is still enigmatic. Increased cytosolic Ca^2+^ arrests mitochondrial trafficking through its binding to Miro1, which dissociates Miro1 from kinesin‐1/Milton/Miro1 complex^50^ or strips kinesin‐1 from the microtubule.^51^ It has been reported that Wld^S^, a protein that slows down Wallerian degeneration of axons, exerts its neuroprotection by enhancing mitochondrial trafficking.^52^ Noteworthy, mitochondria in Wld^S^‐expressing mouse brains show stronger Ca^2+^ buffering capacity.^52^ Consistently, axonal mitochondria with lower Ca^2+^ tend to be mobile and increased Ca^2+^ in mitochondrial matrix by the activation of mitochondrial Ca^2+^ uniporter arrests mitochondrial transport in axons.^53^ These results suggest the direct link between axonal mitochondrial mobility and mitochondrial Ca^2+^; however, the details await further investigations.

## 4 MITOCHONDRIAL TRANSPORT CONTRIBUTES TO AXONAL MITOCHONDRIAL QUALITY CONTROL

### 4.1 Anterograde transport for replenishing axonal mitochondria

**Figure 1:**
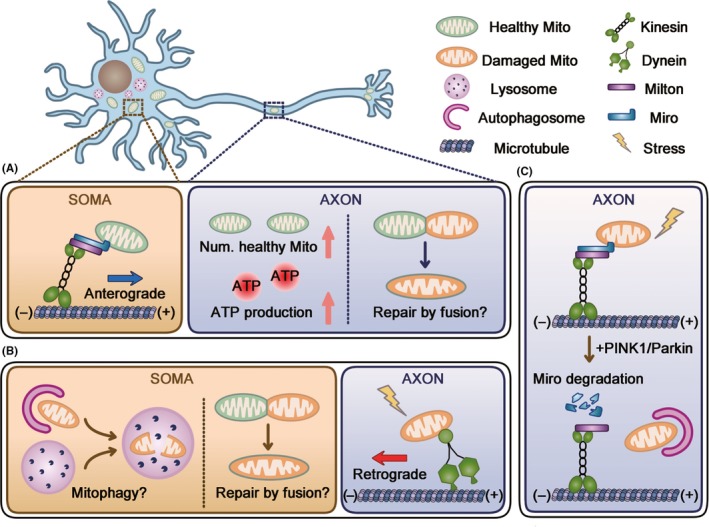
Three possible interplays of mitochondrial transport with mitochondrial quality control in axon. Mitochondria are distributed throughout the whole neuron, and neuronal soma is the main compartment where lysosomes residue and mitochondrial biogenesis takes place. Mitochondrial transport could mediate mitochondrial quality control through the following mechanisms: (A) mitochondria generated in soma are anterogradely transported into axon. Improved axonal mitochondrial quality could benefit from increased number of healthy mitochondria or probably mitochondrial fusion between healthy and damaged mitochondria. (B) Damaged mitochondria undergo retrograde transport for mitophagy in soma or fusing with somatic mitochondria. (C) Damaged mitochondrial mobility is arrested by PINK1/Parkin‐dependent degradation of Miro and cleared by local mitophagy in axon

Although mitochondrial biogenesis is shown to occur locally in the axon,^54^ most new mitochondria are proposed to be generated in the soma.^9^ Supportively, the study using MitoTimer, a time‐sensitive fluorescent protein located in mitochondrial matrix, has shown that the mitochondrial aging is proportional to the distance from soma.^55^ Under such circumstances, mitochondrial anterograde transport is indispensable for mitochondrial quality control by providing young mitochondria to distal regions. Accordingly, enhanced mitochondrial transport contributes to energy recovery from injuries and promotes axon regeneration and neuronal survival.^56^, ^57^ However, it is still unclear whether improved mitochondrial quality in axons rescues axonal injuries merely by enhancing energy production or alternatively by repairing damaged mitochondria through mitochondrial fusion (Figure 1A). Intriguingly, both mitochondrial fusion and fission proteins could interact with mitochondrial transport system.^34^, ^35^ However, the interaction between mitochondrial mobility and dynamics needs to be further addressed.

### 4.2 Retrograde transport for mitochondrial repair or clearance

In addition to the machinery for mitochondrial biogenesis, the degradation organelles, lysosomes, are also predominantly localized in neuronal soma.^58^, ^59^ Thus, it has been speculated that damaged mitochondria in axons return to cell bodies for degradation. Indeed, most mitochondria with low membrane potential are retrogradely transported in intact axons.^27^ Similarly, mitochondria move retrogradely and escape from axons after mild and chronic stress in cultured hippocampal neurons.^43^ In addition, soma‐restricted mitophagy has been shown in both in vitro^60^ and in vivo.^61^ These results indicate that mitochondrial retrograde transport may serve as an axonal mitochondrial quality control strategy by removing damaged mitochondria from axons. However, these studies did not address the final outcome of those mobile mitochondria (being repaired by fusing with somatic mitochondria or being cleared in soma; Figure 1B). Thus, the direct evidence showing axonal mitochondria return to soma for repair or clearance is still lacking.

### 4.3 Arresting mitochondrial mobility to quarantine damages

Accumulating evidence indicates the previously underestimated close association of mitochondrial mobility with mitophagy in axon. PINK1/Parkin, the most extensively studied mitophagy pathway, is found to degrade Miro upon mitochondrial depolarization, which arrests mitochondrial movements.^28^ A more recent study showed that axonal mitochondria were cleared in situ by PINK1/Parkin‐mediated mitophagy.^62^ These observations proposed that the mitochondrial arrest‐and‐mitophagy manner quarantines mitochondrial damages spatially from further affecting the overall mitochondria pool (Figure 1C). However, axonal mitophagy has not been verified in disease‐relevant models and there is a paucity of direct evidence proving the significance of mitochondrial arrest to axonal health. Additionally, Parkin deficiency reduces axonal mitochondrial content in *Drosophila*.^61^ This result is in contrast to the predicted local mitophagy in axons^62^ and implies that soma could be the main compartment for mitophagy in neuron. Further investigations are needed to explore the factors in deciding the intracellular sites where axonal mitochondria degradation occurs. It is possible that mitochondrial transport responds to different stresses and disposes axonal mitochondria in distinct ways.

## 5 IMPLICATIONS OF AXONAL MITOCHONDRIAL TRANSPORT FOR NEUROLOGICAL DISORDERS

### 5.1 Neurodegeneration

Neurodegeneration is characterized by the progressive loss of structure or functions of certain neuronal subtypes, which further leads to functional impairments. Although in the face of increasing clinical demand, there have been few effective therapies for neurodegenerative disorders due to the limited insight into their mechanisms. In recent years, the role of mitochondria in the neurodegeneration has begun to come to prominence.^63^ Here, we focus on the association of mitochondrial transport with neurodegeneration.

#### 5.1.1 Alzheimer's disease (AD)

Alzheimer's disease (AD) is the most prevalent form of dementia worldwide. AD is characterized by progressive death of specific neuronal populations^64^; however, mounting evidence indicates axon degeneration long precedes somatic cell death.^65^, ^66^ One feature of axon pathology in AD is the impaired axonal transport, including axonal mitochondrial transport defects.^67^, ^68^

**Figure 2:**
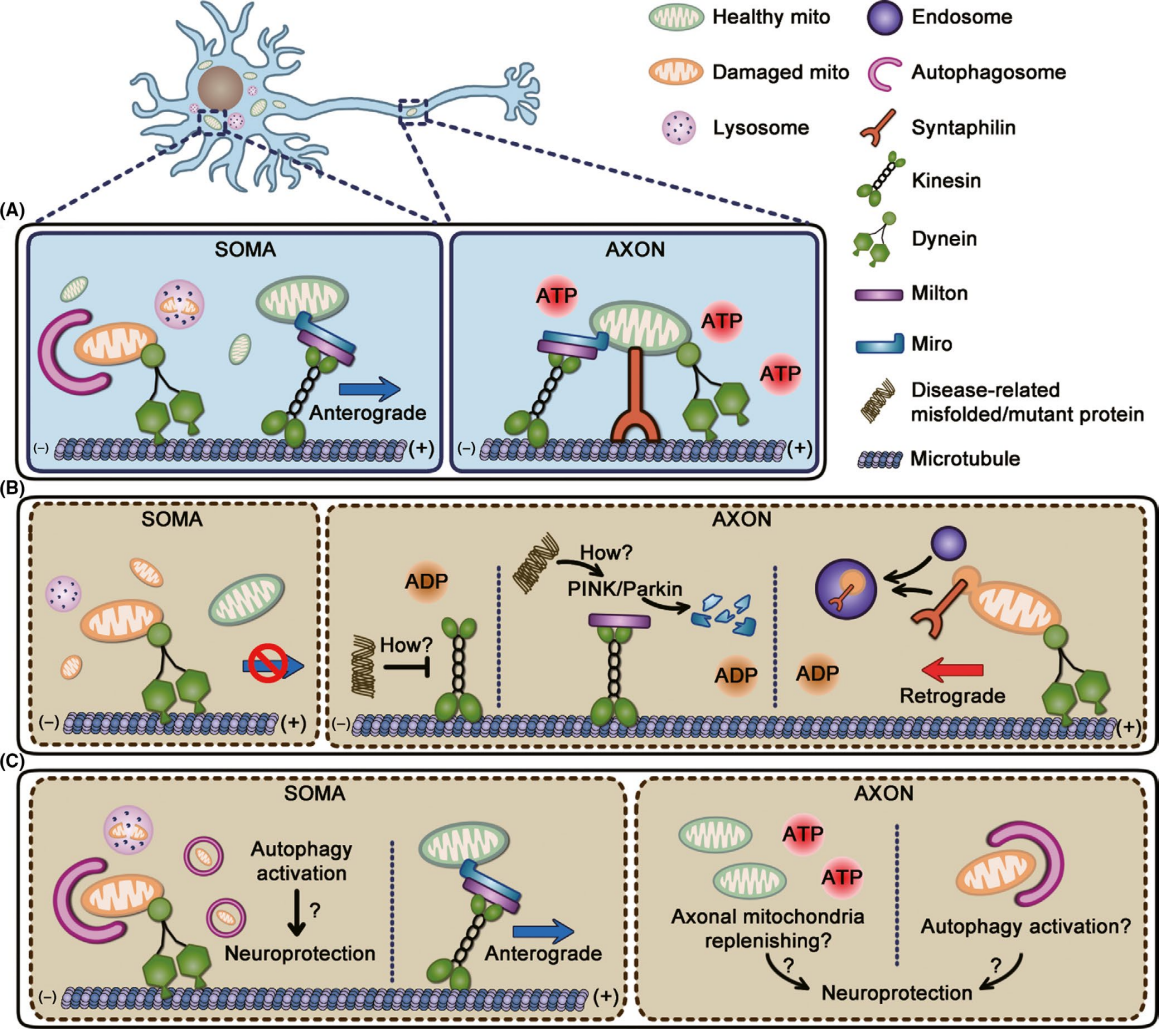
Schematic representation of involvements of mitochondrial transport in neurodegeneration diseases. A, In intact neurons, mitochondrial transport system properly distributes mitochondria which satisfy energy demands of axons. Besides, damaged axonal mitochondria undergo autophagic clearance successfully in soma after retrograde transport. B, Mitochondrial transport is impaired in neurodegeneration diseases. Neurodegeneration‐related misfolded or mutant proteins can inhibit kinesin‐1 activity^71^, ^73^, ^74^, ^103^ and induce degradation of Miro1 in a Parkin‐dependent manner.^89^ The detailed mechanisms underlying the inhibition of mitochondrial anterograde transport in neurodegeneration need further investigations. Additionally, mitochondrial anchor protein syntaphilin buds out of axonal mitochondria and is further degraded in early stages of AD and fALS, which increased mitochondrial retrograde movements in axons.^43^ However, damaged mitochondria accumulate after returning back to soma due to autophagy defects and a lack of mitochondrial anterograde transport in a long run causes axonal energy depletion. C, The combination of corrected mitochondrial trafficking with autophagy activation might confer neuroprotection by degrading both somatic and axonal damaged mitochondria and ameliorating energy stress in axons simultaneously

Extracellular plaques composed of amyloid‐β (Aβ) peptides and intracellular fibrillar tau aggregates are two major histopathological hallmarks of AD. Both Aβ and tau have been shown to disrupt axonal mitochondrial transport. Incubation of cultured hippocampal neurons with Aβ reduces mitochondrial mobility.^10^, ^69^ Unlike Aβ, the impact of tau on axonal transport is still inconclusive under physiological conditions.^69^, ^70^ Tau overexpression in differentiated N2a cells has been shown to arrest axonal mitochondrial trafficking.^71^ However, the inhibitory effect of Aβ on axonal mitochondrial movements is counteracted by tau ablation in cultured hippocampal neurons, indicating a more critical role of tau in mitochondrial trafficking under pathological conditions.^69^ Intriguingly, mitochondrial anterograde transport seems to be more vulnerable in AD.^69^, ^71^, ^72^ One possible mechanism underlying the more obvious loss of mitochondrial anterograde transport is that tau inhibits kinesin‐1 activity but has little effect on dynein‐based movement.^71^, ^73^, ^74^ In addition to mitochondrial anterograde motors, mitochondrial anchor protein syntaphilin buds out of axonal mitochondria and is further degraded in AD‐related mutant hAPP Tg neurons, which increases mitochondrial retrograde movements in axons (Figure 2A,B).^43^ Furthermore, mitochondrial permeability transition pore (mPTP) blockage by genetic depletion of cyclophilin D, one of the structural components of mPTP, attenuates impaired mitochondrial trafficking induced by Aβ and this protective effect is related to the reduced Ca^2+^ and ROS.^75^ These results imply that defective mitochondrial transport in AD may also result from the release of mitochondrial Ca^2+^ and ROS.

The defects of anterograde motor's function together with loss of mitochondrial anchor protein lead to the increase of axonal mitochondrial retrograde transport in AD‐relevant models, which may serve as a first‐line strategy to remove defective mitochondria in axons. However, axonal mitochondria cannot be replenished due to a lack of mitochondrial anterograde transport in a long run. Consistently, reduced mitochondrial content in neurites has been reported in models both in vivo^76^ and in vitro.^71^ Given the critical role of mitochondria in energy production and synaptic Ca^2+^ buffering, impaired axonal mitochondrial transport may lead to synaptic dysfunction and axon degeneration. Furthermore, accumulated mitochondria in soma could not be cleared as a result of autophagy defects (Figure 2A,B),^77^, ^78^ which would further aggravate neuronal death in AD.

#### 5.1.2 Amyotrophic lateral sclerosis (ALS)

Amyotrophic lateral sclerosis (ALS), which is caused by the selective loss of motor neurons in the spinal cord, motor cortex, and brain stem, is the most frequent adult‐onset motor neuron disease.^79^ Approximately 10% of ALS is inherited (familiar ALS, fALS), and several genes have been reported to be related to fALS, including superoxide dismutase 1 (SOD1), TAR DNA‐binding protein (TARDBP; TDP‐43), fused in sarcoma (FUS), vesicle‐associated membrane protein–associated protein B (VAPB), and C9orf72.^80^ Although most ALS cases are sporadic without clear genetic consequences, mitochondrial defects and impaired axonal transport have been shown to be intimately linked with motor neuron degeneration.^81^, ^82^

In fALS‐related animal models or cultured neurons, the alteration of axonal mitochondrial transport is inconsistent with distinct gene mutations. Similar to AD, selective anterograde defect has been reported in cultured cortical neurons expressing ALS mutant SOD1G93A, SOD1A4V, SOD1G85R, SOD1G37R, or VAPBP56S and in embryonic motor neurons expressing SOD1G93A.^12^, ^83^ The intact sciatic nerve of presymptomatic SOD1G93A transgenic mice also exhibits reduced anterograde transport of mitochondria.^84^, ^85^ However, wild‐type TDP‐43 and ALS‐related mutants TDP‐43Q331K, A315T, or M337V lead to decreased mitochondrial mobility in both anterograde and retrograde directions.^85^, ^86^ In addition, the roles of FUS, C9orf72, and their mutants in mitochondrial transport are still conflicting.^87^, ^88^

The molecular mechanisms underlying defective mitochondrial transport remain unsolved. It was reported that ALS mutant SOD1 induced degradation of Miro1 in a Parkin‐dependent manner, which further inhibited anterograde axonal transport of mitochondria.^89^ The degradation of syntaphilin also contributes to the alteration of mitochondrial mobility in the early stages of fALS‐linked mice (Figure 2A,B).^43^ However, given the roles of Miro and syntaphilin in both anterograde and retrograde mitochondrial transport, further investigations are needed to address the selective defects of mitochondrial anterograde transport in some fALS models.

Lack of mitochondrial anterograde transport may result in the loss of mitochondrial content in neurites which has been verified in several fALS models.^12^, ^90^ Thus, increasing mitochondrial mobility might help to replenish axonal mitochondria or to send damaged mitochondria for mitophagy in soma. However, enhanced axonal motility by depletion of syntaphilin fails to slow the disease progression in hSOD1^G93A^ mice.^91^ Instead, improved autophagy‐lysosomal functions by enhancing transport of late endosomes ameliorate ALS‐like phenotype.^92^ Given the mitochondrial dysfunction and autophagy defects in ALS,^93^, ^94^ it may be necessary to combine strategies correcting mitochondrial transport with drugs activating autophagy to improve the mitochondrial quality in ALS (Figure 2C).

#### 5.1.3 Huntington's disease (HD)

Huntington's disease (HD) is an autosomal dominant neurodegenerative disorder caused by the pathogenic expansion of the CAG tract beyond 35 repeats at the N‐terminus of the huntingtin (Htt) protein.^95^ Htt interacts with both anterograde and retrograde transport motor proteins.^96^, ^97^ For certain cargoes, the phosphorylation state of Htt plays a role in deciding the directions of their axonal transport.^99^

Recent studies have indicated that impaired mitochondrial transport involves in the pathology of HD. Mutant of Htt (mHtt) impairs mitochondrial trafficking in both directions in neurons.^100^, ^101^ Noteworthy, in striatal neurons, mitochondrial transport impairment occurs before mHtt aggregates formation^102^ while mitochondria become immobile adjacent to aggregates in cortical neurons,^101^ suggesting the vulnerability of striatal neurons to mHtt toxicity compare with other neuronal subtypes.

The mechanism by which mHtt inhibits mitochondrial transport is not fully understood. Soluble N‐terminal mHtt interacted with mitochondria, which interferes with the association of microtubule‐based transport motors with mitochondria.^102^ Alternatively, mHtt could sequester motor proteins into aggregates and abolish axonal mitochondrial trafficking in neurons.^103^

#### 5.1.4 Parkinson's disease (PD)

Parkinson's disease (PD) is characterized by the selective loss of dopaminergic neurons in the substantia nigra and intracellular inclusions containing aggregates of α‐synuclein.^104^ Although mitochondrial dysfunction is generally accepted underlying PD pathogenesis, the alteration of mitochondrial transport in PD neurons is still inconclusive. Besides the aforementioned studies on Parkin or PINK1, two PD‐linked proteins, some other evidence emphasizes the involvement of mitochondrial transport in PD pathology. 6‐OHDA and MPP^+^, two toxins causing dopaminergic neuron death, disrupt axonal mitochondrial transport in cultured neurons, and this inhibitory effect on mitochondrial transport can be reversed by antioxidants,^105^, ^106^ indicating ROS may be one of the consequences of mitochondrial arrest in PD‐related models.

### 5.2 Neuropsychiatric disorders

Mitochondrial dysfunctions and mitochondrial motility defects have also been associated with several psychiatric disorders.

Although mitochondrial oxidative phosphorylation system (OXPHOS) deficits, especially complex I deficit, contribute to schizophrenia,^107^ direct evidence for altered mitochondrial trafficking in schizophrenia has been lacking. Disrupted in schizophrenia 1 (DISC1) is a genetic risk factor for schizophrenia.^108^ DISC1 interacts with TRAK1 and Miro1 to regulate anterograde transport of mitochondria, which is impaired by a rare DISC1 sequence variant.^109^ In addition, NDE1 and GSK3β, two interactors of DISC1, also function with TRAK1 to regulate axonal mitochondrial motility.^110^ DISC1 may also collaborate with syntaphilin to anchor mitochondria in axons.^111^ In neuronal stress, DISC1 dysfunction impairs mitochondrial functions.^112^ However, it remains unclear whether DISC1‐related mitochondrial trafficking plays a role in mitochondrial dysfunction in schizophrenia.

The cannabinoid CB1 receptor may play a critical role in the pathology of various neuropsychiatric disorders including alcoholism, depression, anxiety, and schizophrenia.^113^ Interestingly, CB1 receptor localizes to mitochondria where its activation inhibits mitochondrial respiration and mobility, which contributes to acute cannabinoid‐induced memory impairment.^114^

Depression was found to be associated with impaired mitochondrial quality indicated by increased production of mtROS.^115^ Depression can be attributed to a loss of serotonin which increases mitochondrial motility in cultured hippocampal neurons.^116^ More intriguingly, dopamine, another depression‐related neurotransmitter, seems to exert different impacts on mitochondrial trafficking through multiple dopamine receptors.^117^

Current evidence has implied the involvement of both mitochondrial dysfunctions and transport alternations in neuropsychiatric disorders. However, it remains enigmatic how mitochondrial dysregulations may contribute to these diseases. Since the neurotransmitter receptors are promising therapeutic targets for not only psychiatric disorders but also other neurological diseases,^118^, ^119^ their impacts on axonal mitochondrial quality and mobility need to be addressed in details. Further researches in this field will extend our knowledge about the pathology of psychiatric disorders and open a way to develop novel therapies.

### 5.3 Traumatic axonal injury

**Figure 3:**
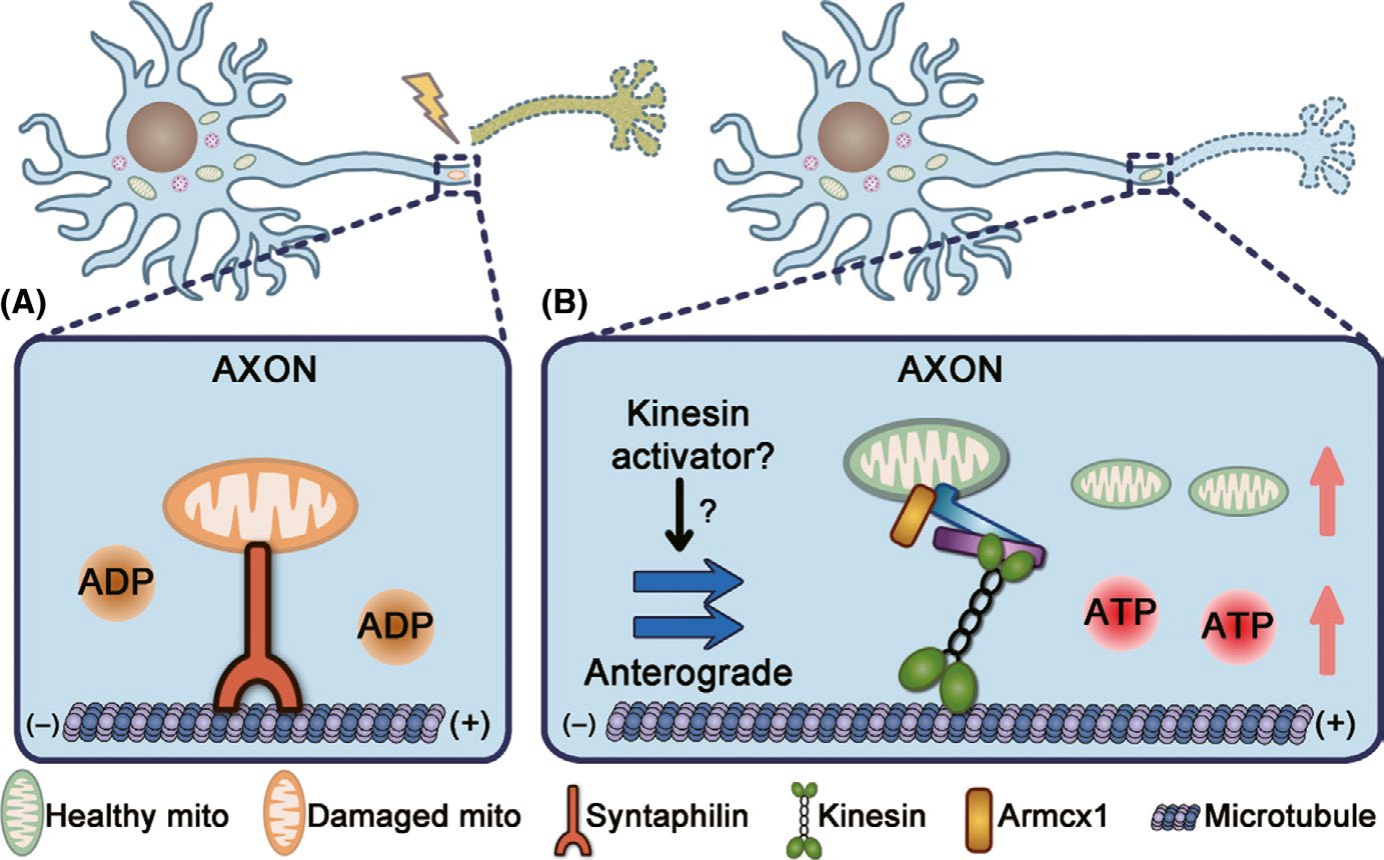
Schematic representation of neuroprotection of mitochondrial anterograde transport against TAI. A, TAI causes mitochondrial damages and ATP depletion in injured axons of mature neurons in which syntaphilin expression is higher and mitochondrial mobility is limited. B, Increased mitochondrial anterograde transport by syntaphilin knockout^56^ or overexpression of Miro1,^56^, ^122^ DLK‐1,^122^ or Armcx1^57^ replenishes axonal mitochondria and rescues energy collapse in injured axons, which further promotes axon regeneration. It is worth further investigations to treat TAI by enhancing mitochondrial anterograde transport with kinesin activators

An estimated 50 million people will experience traumatic brain injury (TBI) every year, which has become a global health concern.^120^ Traumatic axonal injury (TAI), as an important pathoanatomical subtype of TBI, is a major cause of mortality and functional impairment.^121^ Lines of evidence suggest that mitochondrial transport plays a critical role in TAI by using axotomy. Axotomy causes acute mitochondrial membrane potential loss and ATP depletion in injured axons of mature neurons in which syntaphilin expression is higher and axonal mitochondria are less mobile^56^ (Figure 3A). Increased mitochondrial transport by either Miro1 overexpression or syntaphilin knockout promotes axonal regeneration by replenishing healthy mitochondria and thus rescuing energy deficits in injured axons^56^ (Figure 3B). Additionally, dual‐leucine zipper kinase‐1 (DLK‐1), a regulator of axon regeneration, elevates axonal mitochondrial density in a Miro‐independent manner, and increased axonal mitochondria are required for axon regeneration after axotomy.^122^ Armadillo repeat–containing, X‐linked 1 (Armcx1) could interact with Miro1 and enhance mitochondrial transport in adult retinal ganglion cells (RGCs).^57^ Armcx1 overexpression promotes mitochondrial transport and neurite outgrowth in cortical neurons (Figure 3B) and, more importantly, promotes neuronal survival and axon regeneration after optic nerve injury in vivo.^57^ Taken together, mitochondrial transport resolves bioenergetic crisis in injured axons by replenishing axonal mitochondria, suggesting that modulating mitochondrial transport might provide a novel strategy to axonal regeneration in TAI.

## 6 PERSPECTIVE

Mitochondrial transport is critical to maintain the healthy state of mitochondrial population in axons and play an important role in various neurological and psychiatric disorders. However, many issues in this field need to be addressed. It is unknown how motor and anchor proteins sense mitochondrial quality and make corresponding respond. Besides, the intracellular sites where axonal mitochondria undergo mitophagy await to be untangled. It is also worthwhile to investigate how mitochondrial transport coordinates with other mitochondrial quality control strategies, such as mitophagy and mitochondrial fusion/fission. Fortunately, emerging technologies, such as optogenetics,^26^, ^123^ have implemented more precise control of mitochondrial quality and mitochondrial motor or anchor proteins, which will help to extend our understanding of how mitochondrial transport involves in mitochondrial quality control in axon. Although the defects of axonal mitochondrial trafficking have been implicated in various neurological disorders, limited efforts have been made to rescue impaired mitochondrial transport in disease‐relevant models. In addition, mitochondrial transport must work in tandem with other mitochondrial quality control strategies to maintain axonal mitochondrial homeostasis. Thus, the combination of corrected mitochondrial trafficking with enhanced mitochondrial biogenesis or mitophagy (Figure 2C) may be indispensable for the treatment of neurological disorders. On the other hand, mitochondrial trafficking may serve as a novel readout in screening potential compounds for neurological disorders. In particular, mitochondrial anterograde transport seems to be a promising therapeutic target for TAI and the activator of kinesin‐1 has been available (Figure 3B).^124^ Taken together, pioneer investigations are needed to explore the translational implications of axonal mitochondrial transport in central nervous system disorders.

## CONFLICT OF INTEREST

The authors declare no conflict of interest.
